# Hippocampal atrophy but not white-matter changes predicts the long-term cognitive response to cholinesterase inhibitors in Alzheimer’s disease

**DOI:** 10.1186/s13195-015-0155-9

**Published:** 2015-11-23

**Authors:** Yu-Wen Cheng, Ta-Fu Chen, Ting-Wen Cheng, Ya-Mei Lai, Mau-Sun Hua, Ya-Fang Chen, Ming-Jang Chiu

**Affiliations:** Department of Neurology, National Taiwan University Hospital, College of Medicine, National Taiwan University, No. 7, Chung-Shan S Rd., Taipei, 100 Taiwan; Department of Psychology, College of Science, National Taiwan University, Taipei, Taiwan; Department of Medical Imaging, National Taiwan University Hospital, College of Medicine, National Taiwan University, No. 7, Chung-Shan S. Rd., Taipei, 100 Taiwan

## Abstract

**Introduction:**

This study aimed to investigate the feasibility of predicting the long–term effects of cholinesterase inhibitors (ChEI) with common clinical neuroimaging parameters of Alzheimer’s disease, including medial temporal lobe atrophy (MTA) and white matter hyperintensity (WMH).

**Method:**

A cohort of 353 patients with very mild to moderate Alzheimer’s disease received cholinesterase inhibitors and were followed for a median of 46.6 months. Baseline clinical data, including age, educational level, Clinical Dementia Rating (CDR), Taiwanese Mental State Examination (TMSE), and visual scoring for MTA and WMH were tested as possible predictive factors that influence the survival from a TMSE decline of at least 3 points.

**Results:**

During the follow-up period, 162(46 %) patients had a significant TMSE decline. Patients with age-adjusted prominent MTA had a significantly shorter TMSE-decline free interval than those without (43.4 ± 4.5 months vs. 68.2 ± 9.5 months, log rank test p-value =0.001). However, the severity of WMH does not significantly influence cognitive outcomes. Cox regression analysis identified that younger age at the time of starting ChEI (p < 0.0005) and higher total MTA scores (p = 0.002) predict a more rapid TMSE decline under ChEI therapy.

**Conclusions:**

Younger age at the time of starting ChEI and higher visual scoring of MTA may imply a more advanced Alzheimer’s pathology. WMH load is not a prognostic indicator of treatment response to ChEI.

## Introduction

Cholinesterase inhibitors (ChEIs) are the only currently available medications that may modestly decrease the cognitive impairment in patients with mild to moderate Alzheimer’s disease (AD) [1–3]. However, the variable response between patients, relatively high long-term costs, and adverse effects make the general application of ChEIs for all patients with early AD unavailable. Several studies have tried to identify clinical and neuroimaging characteristics that might predict the cognitive response to ChEI therapy [4–8]. However, these studies were often limited by their small sample size and short period of follow up.

The medial temporal lobe is known to be the initial site of pathological changes of AD [9]. Previous studies have shown that the degree of medial temporal atrophy (MTA) was associated with the risk of progression from mild cognitive impairment (MCI) to AD [10–12], the disease stage [13], and the rate of cognitive decline [14]. Meanwhile, the association between MTA and the long-term therapeutic response to ChEI is not well established. Another neuroimaging characteristic is the white matter hyperintensity (WMH), which is viewed by some researchers as an indicator of the underlying vascular burden that contributes to cognitive dysfunction [15, 16]. In Taiwan, most of our AD patients who receive ChEI are reimbursed by the National Health Insurance System. A more extensive WMH was one of the exclusion criteria that precluded patients from ChEI paid for by the national insurance; there was a concern that this might indicate an underlying vascular pathology, in addition to Alzheimer’s pathology [17]. Recent studies have shown an increased burden of WMH with an increasing severity of AD [18, 19]. However, whether or not the presence of WMH would influence the cognitive response to ChEI therapy remains undetermined.

We aimed to identify common neuroimaging indicators for the cognitive outcome predictors in AD patients who received ChEI therapy.

## Method

### Patient recruitment

We recruited patients with very mild to moderate AD, Clinical Dementia Rating (CDR) scored 0.5 ~ 2, who received both cholinesterase inhibitor therapy and regular follow-up at the memory clinic of National Taiwan University Hospital, from August 1999 to June 2012. The diagnosis of AD was made after a comprehensive history review, neurological examination, laboratory survey, and neuroimaging study. All of the patients met the diagnostic criteria for probable AD dementia proposed by the National Institute of Neurological and Communicative Disorders and Stroke and the Alzheimer’s Disease and Related Disorders Association (NINCDS-NDRDA) work group in 1984 [20]. Before starting the cholinesterase inhibitor, clinical data were collected prospectively, including basic demographic data, education level, baseline TMSE (Taiwanese Mini-Mental State Examination) score [21, 22], and CDR. A standardized regular follow up of TMSE and CDR was carried out before the trial, six months after, and annually after starting the cholinesterase inhibitor. Patients who were lost to follow-up within six months of cholinesterase use (seven patients, 2 % of patients initially recruited) were excluded, given that there were no clinical data available for evaluation of their response to ChEI. The ChEIs used in this cohort included donepezil (n = 240, 68 %), rivastigmine (n = 59, 17 %), and galantamine (n = 54, 15 %). The dosages of ChEIs were titrated up to the standard daily dose of 5 to 10 mg for donepezil, 6–9 mg for rivastigmine, and 16 mg for galantamine (if tolerable); changing from one ChEI to another was allowed if intolerable side effects were encountered.

### Image acquisition

Brain computed tomography was performed using one of our multi-detector CT scanners, including GE LightSpeed 16, VCT (GE Healthcare, Milwaukee, WI, USA), SOMATOM Sensation 64, and Emotion 16 (Siemens Medical Solutions, Erlangen, Germany). Axial cuts were taken at the top of the C1 lamina through the top of the calvarium. Axial images were reconstructed with 5-mm-thick sections at 5-mm intervals. In some of our subjects, coronal reconstructed images of 5 mm thickness were also available.

Magnetic resonance imaging (MRI) of the brain was performed on a 1.5TMR unit, either GE Excite (GE Healthcare) or MAGNETOM Sonata (Siemens Healthcare). The scanning protocol included axial FLAIR, fast spin-echo T2-weighted sequences, and a coronal T1WI or a 3D T1WI. The axial slices were positioned to run parallel to a line that joins the most inferoanterior and inferoposterior parts of the corpus callosum and had a thickness of 5 mm with a gap of 1.5 mm.

### Visual scoring for medial temporal atrophy and white matter hyperintensity

MTA was rated on a coronal view of brain CT or T1-weighted MRI, using the scoring system proposed by Scheltens *et al.* [23]. Each side of the medial temporal lobe and hippocampus was scored from 0 to 4. A higher MTA score indicated a higher degree of medial temporal lobe atrophy.

As the correlation of aging and MTA has been well established, we defined prominent MTA according to the patient’s age upon recruitment. A prominent MTA was defined as a MTA score more than 1 on either side for patients 75-years-old and under. For patients older than 75 years, a MTA score more than 2 was required to define a prominent MTA.

To determine the extent of WMH, a visual scoring system modified from that suggested by Fazekas *et al.* [24] was used. Each brain CT or MRI scan of the FLAIR series was rated from 0 to 3 on a coronal view, across two brain areas: frontal and parietal lobes of either side. The total WMH was scored 0 ~ 12, with the higher score indicating more extensive white matter hyperintensity. For WMH scoring, there is generally no accepted cut-off-point for different age groups. We defined a significant WMH if a brain area had a WMH score of 2 or above. The participants were stratified according to the severity of WMH, which included: (1) the presence of a significant WMH in any of the four brain areas; (2) the number of brain areas with significant WMH; and (3) the summation of the WMH scores in all four brain areas.

To determine the inter-rater reliability of visual scoring, 20 MRI or CT scans were rated for MTA and WMH scoring by two independent raters (YFC and YWC) who were blinded to the clinical data. The inter-rater reliability study showed good agreement between raters for both visual scoring for MTA (intra-class correlation coefficient = 0.954) and WMH (intra-class correlation coefficient = 0.950).

The study was approved by the National Taiwan University Hospital’s Institutional Review Board. All investigations were conducted according to the principles expressed in the Declaration of Helsinki. We obtained written informed consent from the participants or their proxies.

### Statistical analysis

We used the Pearson chi-square test to compare the between-group differences of categorical variables and the Mann Whitney *U* test for numerical variables. The Log rank test was used for survival data. Stepwise linear regression analysis was performed separately to evaluate factors associated with a significant MTA and WMH. Inter-rater reliability for the visual ratings of WMH and MTA were measured by intra-class correlation coefficients. To access the cognitive response to cholinesterase inhibitors, we performed a survival analysis; we plotted the Kaplan-Meier survival curve by setting the endpoint as a significant TMSE decline from the baseline of more than 2 points. Patients who passed away or were lost to follow-up before a clinical evaluation at six months were excluded. A Cox proportional hazard model was applied to analyze factors affecting the time until a significant TMSE decline. Data were analyzed using the statistical software PASW for windows, version 18.0.

## Results

Three hundred and fifty-three patients (217 women and 136 men) with very mild to moderate AD were included in this study. The median duration of follow up was 46.6 (7.47 ~ 157.4) months. The mean age of the population was 74.1 ± 10.3 years with a mean education level of 8.9 ± 5.1 years; their mean baseline TMSE score was 19.7 ± 4.1 points and mean CDR score was 0.5 in 195 (55 %) patients, 1 in 129 (36 %) patients, and 2 in 19(5 %) patients (Table 1).

**Table 1:** Demographic data and neuroimaging characteristics of the subjects

Baseline characteristics	Mean ± SD, or number (%)
Age of starting ChEI (year)	74.1 ± 10.3
Gender, female/male, No. (%)	218/135 (62/38)
Education (year)	8.9 ± 5.1
Baseline TMSE	19.7 ± 4.1
Baseline CDR, No. (%)	
0.5	195 (55)
1	129 (36)
2 ~ 3	19 (5)
Total MTA score	3.4 ± 1.7
Maximum MTA score, No. (%)	
0	23 (7)
1	86 (24)
2	174 (49)
3	61 (17)
4	9 (3)
Prominent MTA adjusted by age, No. (%)	136 (39)
Total WMH score	3.6 ± 3.1
Total WMH score, No. (%)	
0 ~ 3	165 (47)
4 ~ 7	125 (35)
8 ~ 12	64 (18)
Number of areas with WMH score > =2	
0	237 (67)
1 ~ 2	49 (14)
3 ~ 4	67 (19)

*CDR* Clinical Dementia Rating, *ChEI* cholinesterase inhibitor, *MTA* medial temporal atrophy, *TMSE* Taiwanese Mini-Mental State Examination, *WMH* white matter hyperintensity

Patients were followed until the study end-point, death, or loss to follow-up. In summary, 162 (45.8 %) patients reached an end point (a more than two-point TMSE decline), 10 (2.8 %) died, and 65 (8.4 %) were lost to follow-up before a significant TMSE decline.

The mean survival time from a significant TMSE decline of all patients was 56.0 ± 5.2 months. The Kaplan-Meier survival curve for the TMSE decline did not differ significantly between patients with or without a significant WMH (a dichotomy), defined as a WMH score of two or more points at any brain area (log rank test p-value = 0.540). There were no significant differences in the survival curves between patients stratified by a total WMH dichotomy of 0–3 versus 4–12 (log rank test p-value = 0.431); there was no significant difference among the three groups, comprising a score of 0 to 3, 4 to 7, and 8 to 12 (log rank test p-value = 0.514); in addition, there was no significant difference among patient groups stratified by number of areas (a trichotomy) with significant WMH of 0, 1 ~ 2, or 3 ~ 4 areas (log rank test p-value = 0.142).

**Fig. 1:**
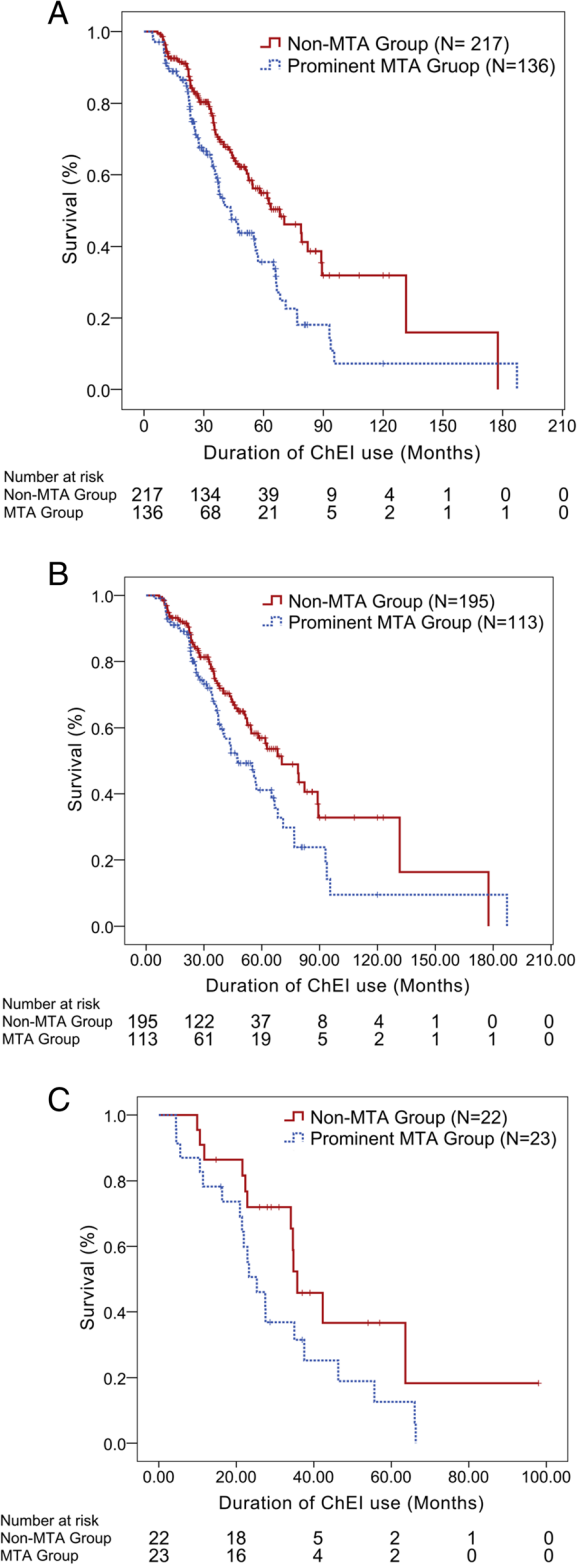
TMSE decline-free survival under ChEI therapy. The Kaplan-Meier survival curves were plotted by setting the endpoint as a significant TMSE decline from the baseline for more than 2 points, for **a** all patients (N = 353, log rank test p-value = 0.001), **b** patients 60-years old or older (N = 308, log rank test p-value = 0.019), and **c** patients younger than 60-years old (N = 45, log rank test p-value = 0.071). Prominent MTA predicted shorter TMSE-decline free survival for patients 60-years old or older when starting ChEI therapy. *TMSE* Taiwanese Mini-Mental State Examination, *ChEI* cholinesterase inhibitor

On the other hand, patients with a prominent MTA had a shorter TMSE decline-free survival under treatment with a ChEI (Fig. 1a, log rank test p-value = 0.001). The median survival time from significant TMSE decline was 68.2 ± 9.5 months for patients without prominent MTA (n = 217, 61 % censored), compared with 43.4 ± 4.5 months for patients with prominent MTA (n = 136, 43 % censored). When we exclude those patients who were lost to follow up during the study period, the remaining 278 patients still showed a significantly shorter TMSE decline-free survival for patients with prominent MTA (log rank test p-value < 0.0005).

To illustrate the effect of individual demographics and neuroimaging characteristics as risk factors on TMSE decline-free survival, we performed forward stepwise Cox regression analysis for independent variables; the variables included age of starting ChEI, gender, educational level (by year), baseline TMSE, CDR, total MTA score, and total WMH score. Younger age at the time of starting ChEI (p < 0.0005) and higher total MTA score (p = 0.002) predicted a more rapid decline of TMSE while receiving ChEI therapy (Table 2). The total WMH score did not significantly predict the TMSE decline-free survival.

**Table 2:** Regression coefficients in Cox proportional hazard models of factors associated with TMSE decline-free survival

Model		B	SE	Exp(B) (95 % CI)	P-value
Model I	Age of starting ChEI	−0.030	0.008	0.971 (0.955 ~ 0.986)	<0.0005
Model II	Age of starting ChEI	−0.035	0.008	0.965 (0.949 ~ 0.981)	<0.0005
	Total MTA score	0.147	0.047	1.158 (1.056 ~ 1.271)	0.002

*CDR* Clinical Dementia Rating, *ChEI* cholinesterase inhibitor, *MTA* medial temporal atrophy, *TMSE* Taiwanese Mini-Mental State Examination, *WMH* white matter hyperintensity, *CI* confidence interval; excluded variables in Model II: gender, educational level, baseline TMSE, baseline CDR, and total WMH score; excluded variables in Model I: gender, educational level, baseline TMSE, baseline CDR, total WMH score, and total MTA score

**Fig. 2:**
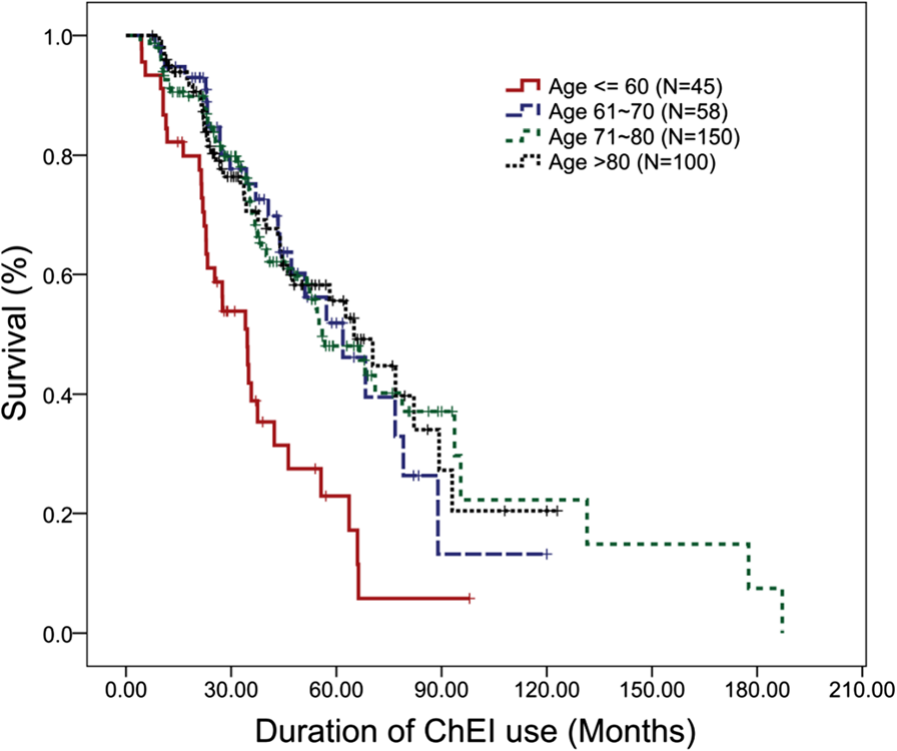
TMSE decline-free survival between patients stratified by different age groups. Patients who started ChEI therapy at the age of 60 years or younger had a more rapid TMSE decline as compared to other age groups *TMSE* Taiwanese Mini-Mental State Examination, *ChEI* cholinesterase inhibitor

Because age was a significant confounding factor that strongly predicted TMSE decline-free survival, we compared the Kaplan-Meier survival curves between different age groups. We found that the survival curves differ significantly for patients 60-years old or less from those older than 60 years (Fig. 2, log rank test p-value < 0.0005). We performed subgroup analysis, according to age, in order to compare the significance of WMH and MTA in separating the Kaplan-Meier survival curve. The severity of WMH did not separate the survival curve significantly in patients either younger or older than 60-years old. On the other hand, a prominent MTA consistently predicted shorter TMSE-decline free survival (Fig. 1b, log rank test p-value = 0.019) for patients 60-years old or older (n = 308). The median survival time was 70.4 ± 7.9 months for patients without prominent MTA (n = 195, 63 % censored), compared with 47.4 ± 6.2 months for patients with prominent MTA (n = 113, 49 % censored). For younger patients (n = 45), a similar trend was present (Fig. 1c, log rank test p-value = 0.071). In patients 60-years old or younger, the median survival time was 35.7 ± 4.2 months in patients without MTA, compared with 25.3 ± 3.5 months for those with significant MTA.

We performed a multiple stepwise linear regression analysis by using the total MTA score or total WMH score as dependent variables to separately identify their respective predictive factors. The examined independent variables included the patient’s age, educational level, baseline CDR, baseline TMSE, and total WMH score or total MTA score. We found that a higher total WMH score, higher educational level, and lower TMSE are associated with a higher total MTA score (Table 3). On the other hand, an older age (p < 0.0005), a higher total MTA score (p < 0.0005), and a higher baseline CDR score (p = 0.003) are associated with a higher total WMH score (adjusted R^2^ 0.276). We also examined the effect of vascular risk factors on the total WMH and MTA scores. We used age, education level, hypertension (n = 265), diabetes mellitus (n = 266), hyperlipidemia (n = 260), and obstructive sleep apnea (n = 228, represented by snoring) as independent variables in multiple stepwise linear regression analyses. In addition to older age (p < 0.0005), we found only the presence of diabetes mellitus (p = 0.007) was positively associated with the total WMH score (adjusted R^2^ 0.186). However, no vascular risk factor was associated with the total MTA score.

**Table 3:** Regression coefficients in linear regression models of factors associated with total MTA score

Model		B (95 % CI)	β	P-value	Adjusted R^2^
I	Total WMH score	0.202 (0.147 ~ 0.258)	0.361	<0.0005	0.128
II	Total WMH score	0.181 (0.124 ~ 0.237)	0.323	<0.0005	0.150
	Baseline CDR	0.728 (0.275 ~ 1.182)	0.162	0.002	
III	Total WMH score	0.186 (0.130 ~ 0.242)	0.332	<0.0005	0.173
	Baseline CDR	0.822 (0.371 ~ 1.273)	0.183	<0.0005	
	Education (year)	0.053 (0.020 ~ 0.086)	0.160	0.001	
IV	Total WMH score	0.187 (0.133 ~ 0.242)	0.334	<0.0005	0.207
	Baseline CDR	0.406 (−0.028 ~ 0.894)	0.090	0.102	
	Education (year)	0.086 (0.051 ~ 0.122)	0.260	<0.0005	
	Baseline TMSE	−0.099 (−0.149 ~ −0.050)	−0.240	<0.0005	
V	Total WMH score	0.197 (0.143 ~ 0.250)	0.351	<0.0005	0.203
	Education (year)	0.089 (0.053 ~ 0.125)	0.268	<0.0005	
	Baseline TMSE	−0.117 (−0.162 ~ −0.072)	−0.282	<0.0005	

*CDR* Clinical Dementia Rating, *ChEI* cholinesterase inhibitor, *MTA* medial temporal atrophy, *TMSE* Taiwanese Mini-Mental State Examination, *WMH* white matter hyperintensity, *CI* confidence interval; Variables entered into stepwise linear regression analysis: age of starting ChEI, educational level, baseline TMSE, baseline CDR, and total WMH score

## Discussion

In this study, we founded that: (1) for patients with mild to moderate AD, a lower total MTA score and an older age predicted a better long term cognitive response to ChEI therapy, defined by a longer TMSE decline-free survival; (2) the severity of WMH does not significantly affect the TMSE decline-free survival; (3) a higher total MTA score was associated with a higher WMH score, higher educational level, and a lower baseline TMSE; and (4) a higher total WMH score was associated with older age, higher total MTA score, and higher baseline CDR.

There are two unique features of this study: first, simple visual scoring for MTA and WMH were used as neuroimaging markers, which are easily applicable in daily clinical practice; second, we used long term TMSE decline-free survival as the endpoint for response to ChEI treatment, rather than short term cognitive improvement, which has been used in most of the previous studies. We set our outcome measurement as the time until a TMSE decline of three or more points and our median follow up time was 46.6 months. Compared with the short -term cognitive response within months, the long-term outcome may be of higher clinical importance for both the patients and the insurance carriers.

In previous studies, volumetric measurements for medial temporal lobe atrophy, hippocampal atrophy, and entorhinal cortex thinning were associated with a poorer cognitive response after ChEI therapy for four or six months [5, 6]. Findings of our study imply that this effect may last for several years (median observation period of approximately four years). Although the volumetric measurement is quantitative and draws less concern regarding inter-rater reliability, the technique is technically demanding and time consuming. This makes this technique not conveniently applicable in routine clinical practice. It was shown by Duara *et al.* that a higher cut-point for MTA score is required for older subjects in order to separate AD patients from age-matched controls [25]. In the original study of Scheltens *et al.* [24], a visual scoring of two or more successfully separated AD patients from age-matched controls; the median age of the study participants was 72.8 years. In another study by Duara *et al.* [26], the authors reported a mean MTA score of 2.45 on the left side and 2.43 on the right side in a group of 53 AD patients who had a mean age of 79.9 years. In this study, we define a “prominent MTA” as a MTA score of three or more for patients older than 75 years and a MTA score of two or more for patients 75 years old or younger. This age-adjusted cut-off-score for defining “prominent MTA” subsequently performed better in separating the TMSE decline-free survival curve under ChEI treatment. This finding is concordant with previous studies using volumetric measurements; this validates the visual scoring for MTA measurement as a marker for the rate of TMSE decline.

Unlike MTA, the role of white matter change in disease progression and response to ChEI therapy for AD patients remains controversial [27]. In a study by Connelly *et al.*, the presence of both hypertension and white matter lesions was associated with a less favorable functional outcome after ChEI therapy for six months [7]. Other studies failed to show a significant correlation between the severity of WMH and cognitive or functional outcome after ChEI therapy for three months [4], one year [28], or nine months or more [8]. On the other hand, when considering the subcortical hyperintensities in the cholinergic pathways using the Cholinergic Pathways Hyperintensities Scale (CHIPS), Behl *et al.* showed that patients in the high CHIPS group had improvement in executive functioning and working memory tasks after receiving ChEI therapy for one year [28]. Our study enrolled a large sample size and had a relatively long follow-up period; we observed no significant correlation between the severity of WMH and ChEI therapeutic responses, in terms of the TMSE decline-free survival. Regarding the global white matter lesion burden, the WMH scoring cannot differentiate an underlying pathological change or anatomical involvement. For patients with AD, the white matter changes may be part of the subcortical changes secondary to the underlying neurodegenerative process [29]; alternatively, it may be related to concurrent vascular disease or vascular risk factors [30–32]. In order to exclude the possible confounding effect of vascular risk factors on the total WMH and MTA scores, we used hypertension, hyperlipidemia, diabetes mellitus [31], and obstructive sleep apnea [32] as independent variables in multiple stepwise linear regression analyses and found that in addition to old age, diabetes mellitus is the only risk factor that was related to total WMH scores. The heterogeneous pathophysiology underlying global white matter change may be an important reason why the WMH rating was less effective in predicting outcomes. Rating the subcortical involvement of the cholinergic pathway may have more of a clinical implication, although it may be less applicable in daily clinical practice [8, 28].

Linear regressions showed that the severity of MTA was positively associated with the severity of WMH and educational level; it was negatively associated with baseline TMSE. The association of WMH with MTA was independent of age, TMSE, or CDR. This supports the possible common neurodegenerative process that underlies both MTA and WMH. Another interesting finding was the association between the severity of MTA and educational level. Education has been shown to be a protective factor against the development of MCI and AD in several epidemiological and longitudinal observational studies [33, 34]. The cognitive reserve theory suggests that cognitive activities associated with education, occupational attainment, or leisure may delay the clinical expression of AD pathology by increasing the neuronal reserve or neuronal compensation [35]. In our study, a higher education level was associated with higher MTA scores. This finding is consistent with the cognitive reserve theory that more advanced neurodegenerative pathology may be present in patients with higher educational levels than those with lower educational level with the same clinical severity. A negative association between education and regional cerebral blood flow in the parietotemporal area [36] or gray matter densities in the hippocampal area [37] were also shown in previous studies.

There are some limitations to this study. First, we used the survival time to a significant TMSE decline, instead of the rate of TMSE decline as the outcome measurement. Because the study subjects are from a cohort that received ChEI therapy reimbursed by the National Health Insurance of Taiwan, most of the patients discontinued ChEI therapy once a significant TMSE decline was observed as there was a discontinuation of support from the insurance system. Nevertheless, the duration before a significant cognitive decline may be more clinically valuable, as this would be more beneficial to prolonging the early stages of AD, considering quality-adjusted life years. Second, we did not control for the type of ChEI used; this allowed for changing from one ChEI to another when intolerable side effects were encountered. A previous meta-analysis showed no evidence of any differences in efficacy between different ChEIs [1]. Clinically, we choose from different ChEIs mainly based on the side effect profile. Thus, the type of ChEI was not controlled when evaluating the cognitive response. Finally, visual scoring for measurement of the severity of MTA and WMH was not qualitative and was associated with a ceiling effect [38]. In addition, there was no generally accepted age-specific cut-off for WMH scoring. The high intra-class correlation coefficient supported good inter-rater agreement in the visual scoring of either WHM or MTA. We also found a consistent association between higher MTA scores and poorer cognitive responses; this was found to be independent of age, TMSE, CDR, or education, using the Cox proportional model for survival analysis. On the other hand, the severity of WMH did not significantly affect cognitive responses; this was true regardless of the fact that we tried variable stratification strategies, including using the maximal score of the areas, the summation of the total scores from all four areas, or the number of areas with WMH. These findings suggest that the global assessment of WMH is of little, if any, value in predicting cognitive outcomes while on ChEI therapy.

## Conclusions

Our results support the application of visual scoring of MTA as an indicator in predicting long term cognitive responses to cholinesterase therapy for patients with mild to moderate Alzheimer’s dementia. On the other hand, the global assessment of the white matter disease burden may not contribute significantly to cognitive outcomes. Furthermore, studies including location or pathway specific neuroimaging characteristics are required to identify possible predictive values of different patterns of WMH in the therapeutic response. At the current stage, we suggest against the application of the global assessment of the white matter disease burden to exclude patients from clinically efficacious therapy under the diagnosis of Alzheimer’s dementia.

## Abbreviations

AD: Alzheimer’s disease
CDR: Clinical Dementia Rating
ChEI: cholinesterase inhibitor
MTA: medial temporal atrophy
TMSE: Taiwanese Mini-Mental State Examination
WMH: white matter hyperintensity
